# Association Between Cognitive Impairment and Poor Oral Function in Community-Dwelling Older People: A Cross-Sectional Study

**DOI:** 10.3390/healthcare13060589

**Published:** 2025-03-07

**Authors:** Yumiko Mishima, Maya Nakamura, Yuhei Matsuda, Keitaro Nishi, Ryota Takaoka, Takahiro Kanno, Toshihiro Takenaka, Takayuki Tabira, Hyuma Makizako, Takuro Kubozono, Mitsuru Ohishi, Tsuyoshi Sugiura, Tatsuo Okui, on behalf of the Collaborative Group

**Affiliations:** 1 Department of Maxillofacial Diagnostic and Surgical Science, Field of Oral and Maxillofacial Rehabilitation, Graduate School of Medical and Dental Sciences, Kagoshima University, Kagoshima 890-8544, Japan; k5782265@kadai.jp (Y.M.); knishi@dent.kagoshima-u.ac.jp (K.N.); k7506163@kadai.jp (R.T.); tokui@dent.kagoshima-u.ac.jp (T.O.); 2 Department of Oral and Maxillofacial Surgery, Faculty of Medicine, Shimane University, Izumo 693-8501, Japan; yuhei@med.shimane-u.ac.jp (Y.M.); tkanno@med.shimane-u.ac.jp (T.K.); 3 Tarumizu Municipal Medical Center, Tarumizu Chuo Hospital, Kagoshima 891-2124, Japan; takenaka@tarumizumh.jp; 4 Department of Occupational Therapy, School of Health Sciences, Faculty of Medicine, Kagoshima University, Kagoshima 890-8544, Japan; tabitaka@health.nop.kagoshima-u.ac.jp; 5 Department of Physical Therapy, School of Health Sciences, Faculty of Medicine, Kagoshima University, Kagoshima 890-8544, Japan; makizako@health.nop.kagoshima-u.ac.jp; 6 Department of Cardiovascular Medicine and Hypertension, Graduate School of Medical and Dental Sciences, Kagoshima University, Kagoshima 890-8544, Japan; kubozono@m.kufm.kagoshima-u.ac.jp (T.K.); ohishi@m2.kufm.kagoshima-u.ac.jp (M.O.); 7 Division of Oral and Maxillofacial Oncology and Surgical Sciences, Tohoku University, Sendai 980-8575, Japan; tsuyoshi.sugiura.b2@tohoku.ac.jp

**Keywords:** cognitive impairment, oral hypofunction, tongue–lip motor function

## Abstract

Background/Objectives: The population of Japan has a high life expectancy, but there is room for improvement in terms of the country’s healthy life expectancy. The long period of care dependency among Japan’s elderly is also a major economic health challenge. Dementia is a major factor in the need for care, and its prevention is a crucial and urgent challenge. There are recent reports of a possible association between changes in oral function and cognitive impairment, but the details of this association remain unclear. To clarify the relationship between poor oral function and cognitive impairment, we conducted an exploratory investigation using a cognitive function assessment (Mini-Cog) administered in a large-scale study and its relevance to oral function. Methods: The study population was 678 community-dwelling individuals aged ≥65 years living in Tarumizu city, Japan, in 2019. Cognitive function was assessed using the Mini-Cog test, and the oral survey was a modification of the content of the Oral Hypofunction Examination as defined by the Japanese Society of Gerodontology. Results: The participants’ median age was 73 years. The oral function results revealed median scores below the oral hypofunction criterion for occlusal force, tongue pressure, oral diadochokinesis, and swallowing function. The results of a binomial logistic regression analysis indicated that tongue–lip motor function was independently associated with oral function in relation to cognitive impairment. Conclusions: The oral function associated with cognitive impairment in this study was tongue–lip motor function. Aiming to improve this function may prevent the exacerbation of cognitive impairment.

## 1. Introduction

The percentage of Japan’s population aged ≥65 years was 29.0% in 2011, and this value is expected to reach 38.7% by 2070. Although the average life expectancy in Japan is long, and often referred to as the country’s “longevity”, there is a significant discrepancy in Japan between the average life expectancy and the healthy life expectancy, with a considerable proportion of the population requiring nursing care for an extended period [1,2,3]. Dementia is the primary cause of such care needs in Japan, and its prevalence is increasing [4]. In 2012, 15% of Japanese people aged ≥65 were diagnosed with dementia, and this figure is expected to reach 20% by 2025 [5]. Japan is thus facing the dual challenges of preventing and counteracting dementia, while also extending healthy life expectancy and improving the population’s quality of life.

Dementia is characterized by a decline in cognitive abilities (including memory impairment) in addition to one or more of the following dysfunctions: loss of speech, loss of action, loss of cognition, and executive function impairment. These impairments result in a noticeable decline in social and occupational functioning, as well as a notable reduction in the individual’s previous level of ability [6]. Although the prevention of memory impairment and the subsequent onset of dementia is undoubtedly a crucial objective, the restoration and maintenance of cognitive functions beyond memory impairment are also considered to be of significant importance. The concept known as mild cognitive impairment (MCI) was introduced relatively recently, in association with the objective of preventing the onset of dementia. MCI is a condition in which an individual’s cognitive function can be restored before the onset of dementia, whereas the symptoms of dementia progress irreversibly once it has developed. This indicates that it is crucial to detect cognitive impairment as early as possible in order to prevent the development of dementia [7,8].

Factors including genetics, social background, education, isolation, and lifestyle contribute to an increased risk of cognitive impairment and dementia [9,10]. Attention has also focused on the link between oral function and cognitive function, with a considerable number of investigations of the relationship between oral dysfunction and cognitive function. For example, Nagatani et al. reported that oral frailty is a risk factor for MCI in community-dwelling older people, and Tanaka et al. observed an association between MCI and tongue pressure [11,12]. One of our research group’s studies also demonstrated that MCI was significantly associated with reduced occlusal force and decreased tongue pressure [13]. However, the majority of these studies used the Mini-Mental State Examination (MMSE), a test of cognitive function, as their primary assessment tool. The MMSE comprises 11 tests and is a straightforward screening instrument based on an internationally recognized questionnaire [14]. Another internationally used cognitive function test is the Mini-Cog, which consists of two tests and is used by many medical institutions because it is quicker and easier to administer than the MMSE and has the same validity as the MMSE [15,16,17,18].

For dental professionals, this research holds significant implications. As frontline healthcare providers who frequently interact with older adults, dentists are uniquely positioned to contribute to dementia prevention efforts. Understanding the relationship between oral function and cognitive health may encourage dentists to adopt a more holistic approach to patient care, recognizing oral health not just as an independent factor but as part of a broader systemic health framework. The aim of this study is to investigate the relationship between oral function decline and cognitive function using the Mini-Cog method, a simpler cognitive function assessment method than conventional methods, in consideration of the increasing need for screening methods for cognitive function.

## 2. Materials and Methods

### 2.1. Participants

**Figure 1:**
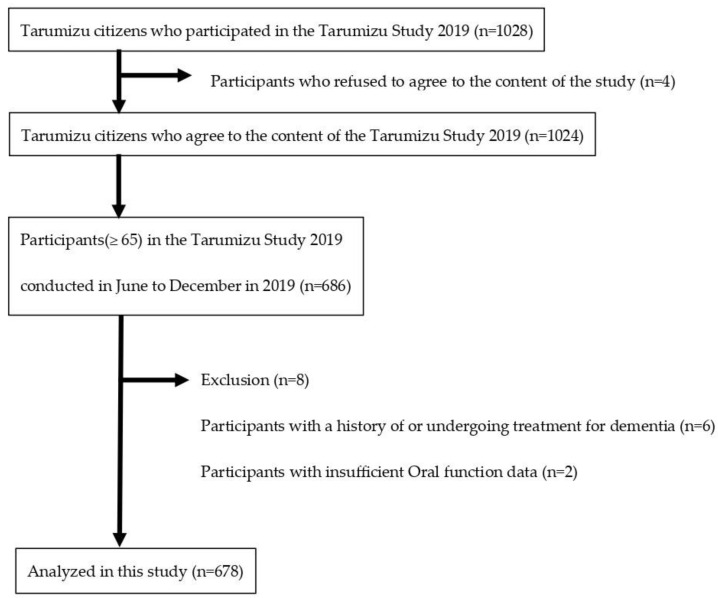
Flowchart for study inclusion.

The study enrollment methodology is depicted in Figure 1. Tarumizu city in the Kagoshima Prefecture has an aging rate of 40%, which is the same as Japan’s aging rate in 2060. For this reason, it was selected as a suitable cohort site and model region for Japan’s future aging society. This cross-sectional study used data from the 2019 Tarumizu Study, a health assessment survey of local residents conducted in cooperation with Kagoshima University, Tarumizu City Hall, and Tarumizu Chuo Hospital. Participants’ eligibility criteria were set as follows: 1. participants were living in Tarumizu city and were recruited by mailed postcards, 2. residents of Tarumizu city who responded would end up participating, with their consent to provide research data, and 3. participants were aged ≥65 years. The exclusion criteria were the following: 1. participants with missing data, 2. participants with pre-existing or undergoing treatment for dementia, and 3. participants with inadequate oral function measurements. The Tarumizu Study was conducted with the approval of the Kagoshima University Hospital Clinical Research Ethics Committee (ref. no. 170351). The participants gave their written informed consent before participating in that study. Cases with missing values for key variables were excluded from the analysis (listwise deletion). No data imputation was performed.

### 2.2. The Mini-Cognitive Assessment Instrument (Mini-Cog^©^)

The Mini-Cognitive Assessment Instrument (Mini-Cog^©^) is a cognitive screening test for older people [15]. The Mini-Cog consisted of two tasks: (1) a three-word recall task and (2) a clock drawing test (CDT). The total score ranged from 0 to 5, calculated as the sum of the recall score (0–3 points) and the clock drawing score (0 or 2 points). Participants scoring ≤ 2 were classified as having cognitive impairment, while those scoring ≥ 3 were classified as typical [19].

For the measurement of the participants’ recall ability, they were first asked to memorize three words presented. After the clock drawing test, the participants were asked to recall the three words again, and the number of words they could say was scored.

For the following score, we asked the participants to draw a standard clock within a 2 min period. Two points were awarded if the participant correctly positioned the numbers and hands of the clock.

### 2.3. Oral Function Measurement

The assessment and diagnosis of the participants’ oral function was based on the diagnostic criteria for oral hypofunction established by the Japanese Society of Gerodontology in 2016, with some modifications to the examination methods [20]. Seven items were assessed: oral hygiene, oral dryness, occlusion force, tongue–lip motor function, tongue pressure, masticatory function, and swallowing function. We used both the data from the oral function survey and the results from the questionnaire for the measurement of oral functions.

#### 2.3.1. Oral Hygiene

We used the tongue coating index (TCI), partially revised, to assess the participant’s oral hygiene [21]. The tongue was divided into three parts (anterior, middle, and posterior), and the degree of tongue coating was assessed visually as three levels (0, 1, and 2). Poor oral hygiene was diagnosed with a TCI score of ≥50% on the tongue.

#### 2.3.2. Oral Dryness

A Moisture Checker (Mucus, Life Co., Saitama, Japan) was used to measure the degree of oral wetness. Mucosal wetness was measured at the center of the dorsum of the tongue, 10 mm from the tip of the tongue [22]. Measurements were taken three times, and the median value was used. Oral dryness was diagnosed if the reading was below 27.0 [23].

#### 2.3.3. Occlusal Force

There are two methods of assessing occlusal force. In the diagnostic criteria for the diagnosis of oral hypofunction, occlusal force can be assessed by measuring a pressure film, which indicates occlusal pressure or using the number of remaining teeth. Gotfredse et al. stated that this force is generally well maintained when >20 teeth remain [24]. In the present study, we used the number of residual teeth and the total number of teeth as indicators of occlusal force, excluding the remaining roots and three mobile teeth. A total score < 20 was categorized as reduced occlusal force.

#### 2.3.4. Tongue–Lip Motor Function

The participants’ tongue–lip motor function was assessed based on oral diadochokinesis (ODK), which comprises the speed and dexterity of the movements of the tongue, lips, and soft palate. Good oral function was defined as pronunciation of all three words (/pa/, /ta/, /ka/) >6x/sec and poor at <6x/sec, assessed using the Kenkou-kun Handy device (Takei Scientific Instruments, Japan) [25]. Participants were instructed to repeat /pa/, /ta/, and /ka/ syllables as quickly and clearly as possible for a duration of 5 s. The average repetition rate (Hz) was recorded. According to the diagnostic criteria established by the Japanese Society of Gerodontology, an ODK rate of <6 repetitions per second in any syllable was classified as impaired tongue–lip motor function [20,26].

#### 2.3.5. Tongue Pressure

Tongue pressure was measured using a JMS Tongue Pressure device (JMS Co., Tokyo, Japan). Each participant was instructed to press their tongue against a disposable balloon probe positioned on the palate. Three repeated measurements were taken, with the highest value recorded. Low tongue pressure was defined as <30 kPa, based on previous research [27].

#### 2.3.6. Masticatory Function

Masticatory function was assessed using a single-item questionnaire: “Are you usually able to chew hard food?” Participants responding “No” were classified as having impaired mastication. While this method has limitations in objectivity, previous studies have validated its correlation with clinical assessments of masticatory performance [28].

#### 2.3.7. Swallowing Function

Swallowing function was assessed by the 10-item Eating Assessment Tool (EAT-10) proposed by the Japanese Society of Gerodontology [29,30]. Each answer to an EAT-10 question was scored from 0 to 4 points, with participants choosing 0 if the answer did not apply to them and 4 if it applied very much to them. An overall score of ≥3 points was considered to indicate possible poor swallowing function. The maximum Eat-10 score was 40 and the minimum was 0. The higher the score, the worse the swallowing function.

All oral and cognitive function assessments were conducted by well-trained dentists and health professionals at the study’s sites.

### 2.4. Covariates

We used information on the participants’ age, sex, education, tobacco smoking history, and social background (e.g., whether the participant lived a solitary life), as well as medical information such as type of medication(s) and medical history. We also used the participants’ body mass index (BMI) and blood pressure (i.e., systolic blood pressure [SBP] and diastolic blood pressure [DBP]) measured on the day of the participants’ visit, and hemoglobin A1c (HbA1c) in the blood sample data.

### 2.5. Statistical Analyses

In our examination of the participant characteristics, continuous variables were expressed as medians and interquartile ranges, and categorical variables were expressed as numbers and percentages. Data normality was tested using the Shapiro–Wilk test. We used the Mann–Whitney U-test for continuous variables and the Chi-square test for categorical variables to examine the association between oral function and cognitive function, and comparisons were made between the two groups of participants with and without cognitive impairment. Candidate confounding factors included those involved in cognitive impairment and investigated in the Tarumizu Study. To assess multicollinearity, Spearman’s correlation coefficients were calculated among all predictor variables. Variables with correlation coefficients ≥ 0.7 were considered collinear and excluded from the regression model to prevent redundancy [31]. In particular, due to high collinearity among oral diadochokinesis measures (/pa/, /ta/ /ka/), only the /pa/ repetition rate was included in multivariate analyses. Multivariate analyses were then performed using the likelihood ratio logistic regression analysis (backward stepwise) of a binomial logistic regression analysis, with the presence/absence of cognitive impairment as the objective variable and a variable without multicollinearity as the explanatory variable. We adjusted for age, sex, BMI, education, tobacco smoking history, number of prescription drugs used, depression, SBP, DBP, and HbA1c. The statistical software JMP^®^ version 14.0 (SAS Institute Inc., Cary, NC, USA, 1989–2022) was used, and *p* < 0.05 was considered significant.

## 3. Results

### 3.1. Characteristics of the Participants

The participants’ characteristics are summarized in Table 1. From the initial 1028 participants, we excluded 4 individuals who did not consent to provide research data. We then focused on participants aged ≥ 65 years. A total of 6 individuals with pre-existing or previously treated dementia and 2 participants with incomplete oral function measurement data were further excluded, resulting in a final sample size of 678 (250 males, 428 females).

The average age of the participants was 73, and the number of women was 427 (63.0%). The results of the oral function measurements revealed that most of the median scores achieved by the participants were within the normal range, as defined by the Japanese Society of Gerodontology’s criteria for oral hypofunction. However, the median scores for occlusal force and ODK/ka/ were below the criteria. About 20% of the participants reported difficulty in chewing hard food, and 10% of the participants’ Mini-Cog scores indicated cognitive impairment.

### 3.2. Association Between Older People with Cognitive Impairment and Measurement Items for Oral Hypofunction

Table 2 presents the results of the group comparisons of oral function with and without cognitive impairment. The participants who had suspected dementia showed significantly poorer occlusal force, ODK/pa/, ODK/ta/, ODK/ka/, tongue pressure, and swallowing function. The median occlusal force value was lower in the participants with cognitive impairment, and that of tongue–lip motor function investigated in oral diadochokinesis was lower in the participants with cognitive impairment in all pronunciations. Among the three pronunciations, the median value for /ka/ was lower than the reference value for oral hypofunction, even in the participants with normal cognitive function. Tongue pressure showed higher median values for the participants with normal cognitive function, and the EAT-10 investigation of swallowing function revealed that, although the value was 0 points in both groups, the interquartile range was wider in the group of participants with normal cognitive function.

### 3.3. Risk Analysis of Poor Oral Function and Cognitive Impairment

The results of each univariate analysis with suspected dementia as the objective variable identified the following as risk factors for dementia: age (odds ratio [OR] 1.1, 95% confidence interval [CI]: 1.06–1.14), sex (OR 2.06, 95%CI: 1.26–3.04), smoking history (OR 1.02, 95%CI: 1.0006–1.032), number of prescription drugs used (OR 1.09, 95%CI: 1.02–1.12), occlusal force (OR 0.96, 95%CI: 0.94–0.98), ODK/pa/ (OR 0.62, 95%CI: 0.49–0.78), ODK/ta/ (OR 0.68, 95%CI: 0.54–0.78), ODK/ka/ (OR 0.63, 95%CI: 0.51–0.79), and tongue pressure (OR 0.96, 95%CI: 0.94–0.99).

The binomial logistic regression analysis showed that sex (OR 1.11, 95%CI: 1.06–1.15), age (OR 2.43, 95%CI: 1.42–4.16), history of depression (OR 4.60, 95%CI: 1.10–19.2), and ODK/pa/ (OR 0.756, 95%CI: 0.59–0.96) were risk factors for dementia.

The results of the multivariate analysis using a binomial logistic regression analysis with cognitive impairment as the endpoint are shown in Table 3.

## 4. Discussion

There is a consensus that oral health has a significant influence on general well-being [32]. In 2016, the Japanese Society of Gerodontology integrated the indicators of poor of oral function and defined a new concept, oral hypofunction [20].

The impact of oral function on overall health is multifaceted. An individual’s oral function status not only has a relatively predictable correlation with the onset of aspiration pneumonia due to poor swallowing ability and masticatory efficiency but also affects impairment in cognitive function [33,34,35,36,37].

Cognitive impairment can also be followed by the onset of dementia. It is estimated that 5–15% of individuals with MCI will develop dementia within a year [8]. Conversely, a return to health has also been observed among individuals with MCI [38,39,40]. Harini et al. also reported that restoration of occlusion by implant overdenture was associated with improved MMSE scores in so-called cognitive abilities [41]. The identification of this MCI state and the postponement of the transition to dementia have become pressing social concerns in the context of an aging population.

A number of indicators are used to diagnose and assess MCI, including the Japanese version of the Montreal Cognitive Assessment (MoCA-J), the MMSE, the revised version of Hasegawa’s Dementia Scale (HDS-R), and the Clinical Dementia Rating (CDR) scale [42]. One of our group’s previous studies demonstrated that oral function was significantly reduced in healthy older adults who were assessed as having MCI by the National Center for Geriatrics and Gerontology Functional Assessment Tool (NCGG-FAT) [13,43]. The commonly employed method is the MMSE, which is utilized as a screening tool for cognitive function by neurologists, geriatricians, and general practitioners in numerous countries [14,44]. The MMSE, comprising 11 questions, was developed by Folstein, is relatively time-consuming (approx. 15 min.), and is susceptible to influence from language and educational levels. In addition, individuals undergoing the MMSE must possess adequate hearing and vision [44,45,46,47].

Recent years have seen the emergence of reports on the utility of cognitive function tests utilizing the Mini-Cog, a test proposed by Borson et al. which assesses five cognitive abilities: sense of direction, immediate memory, calculation ability, short-term memory, and language ability [15,18,48,49,50]. It is a straightforward instrument for identifying cognitive impairment in the elderly [44]. The Mini-Cog is often selected as a cognitive screening tool due to its ease of administration, brevity (requiring only ~5 min), and its demonstrated validity comparable to the MMSE in identifying cognitive impairment (sensitivity: 76–99%, specificity: 83–93%) [15,18,51]. Unlike the MMSE, the Mini-Cog^©^ is less influenced by educational background, making it more suitable for a diverse older population [52].

In the present study, the Mini-Cog assessment indicated that 10.3% of the participants may have had cognitive impairment. This finding is consistent with those of other studies regarding the ratio of healthy elderly individuals suspected of having MCI and is considered appropriate for general social groups [53]. As illustrated in Table 2, the descriptive statistics for healthy elderly individuals and those suspected of having MCI exhibited notable discrepancies in bite force, pronunciation, tongue pressure, and swallowing ability. Nevertheless, our factor analysis with a multivariate analysis revealed no significant discrepancy in bite force, tongue pressure, or swallowing function between the elderly with suspected MCI and those with normal cognition.

Several studies have demonstrated that Mini-Cog may be more effective than MMSE in identifying MCI at an earlier stage [46,51]. In the present study, the evaluation using the Mini-Cog revealed a high incidence of reduced lip movement (as indicated by the pronunciation of /pa/) among older people suspected of having MCI. These findings indicate that early-stage MCI may be preceded by impaired lip movement. Our findings are consistent with the report by Watanabe et al. that a decrease in oral motor ability is an effective indicator for detecting MCI [54].

Dementia patients are known to have a significantly poorer quality of speech [55]. A reduction in lip movement resulting from a decrease in orbicularis oris muscle dexterity has been demonstrated to lead to a reduction in speech clarity and quantity [56]. These findings and our present results indicate that a reduction in the function of the lips may occur at an earlier stage of MCI than a reduction in the function of the tongue and pharynx. In future research, it will be necessary to use sociological or molecular physiological methods to identify the causes of the decrease in lip function that occurs prior to poor performance in other oral functions.

In conclusion, our findings, obtained using pronunciation as an evaluation method, showed a correlation between MCI and oral motor dysfunction, particularly a decrease in lip movement function. These results suggest that elderly people with MCI assessed by the Mini-Cog test may show a decrease in their lip movement ability, and this may be a potential sign of relatively early-stage MCI compared to other poor oral function.

There are some limitations to this conclusion, however. As this was a cross-sectional study, it was not possible to determine which condition occurred first, MCI or oral hypofunction. Further research involving a longitudinal study is necessary. In addition, other research indicated that elderly individuals tend to overestimate their physical functioning without being aware of any poor potential [57]. This is referred to as anosognosia. It is established that anosognosia is a symptom of MCI caused by a reduction in the response of frontal lobe neurons [58]. In light of these findings, we conducted scoring based on subjective evaluation in our study, and it is possible that appropriate results were not calculated for masticatory and swallowing abilities. It is necessary to adapt an objective evaluation method in the future.

## 5. Conclusions

Poor oral function in older individuals is also related to cognitive impairment assessed by the Mini-Cog, which is a simpler and faster method for assessing cognition compared to the MMSE. Our analyses revealed that, among the oral functions tested, lip motor function was associated with cognitive impairment, which suggests that older people with reduced tongue and lip function should be examined for cognitive impairment. This reduction is a reversible factor and could be improved by intervention. The prevention or improvement of the decline of the muscles involved in these functions may prevent the exacerbation of cognitive impairment.

## Figures and Tables

**Table 1:** Demographic characteristics (n = 678).

Item	Category	n, % or Median (IQR)
Age, yrs		73 (69–78.3)
Sex	Male (n)	251 (37.0)
	Female (n)	427 (63.0)
BMI, kg/m^2^		22.9 (20.9–24.9)
Education, yrs		5 (5–6)
Tobacco smoking history, yrs		0 (0–10)
Prescription drugs used, n		3 (1–5)
Solitary life, yes		183 (27.0)
Systemic disease	Stroke (n)	34 (5.0)
	Depression (n)	11 (1.6)
SBP, mmHg		135 (123–146)
DBP, mmHg		77 (70–84)
HbA1c, %		5.6 (5.4–5.9)
Oral function measurements	Oral hygiene (%)	50 (33–83)
	Oral dryness	27.3 (25.4–28.6)
	Occlusal force	19 (8–25)
	ODK repetitions/s /pa/	6 (5.6–6.6)
	ODK repetitions/s /ta/	6 (5.4–6.4)
	ODK repetitions/s /ka/	5.6 (4.8–6.2)
	Tongue pressure (kPa)	33.2 (27.4–38.7)
	Masticatory function	135 (19.9)
	Swallowing function	0 (0–2)
Mini-Cog^©^	Cognitive impairment	70 (10.3)

BMI: body mass index; SBP: systolic blood pressure; DBP: diastolic blood pressure; HbA1c: hemoglobin A1c; IQR: interquartile range; and ODK: oral diadochokinesis.

**Table 2:** Association between cognitive impairment and oral hypofunction measurement items.

Category	n (%) or Median (IQR)		*p*-Value
	Normal n = 608	Cognitive Impairment n = 70	
Oral hygiene (%)	50 (33–83)	50 (33–83)	0.109 ^a^
Oral dryness	27.2 (25.4–28.5)	27.5 (25.5–28.7)	0.649 ^a^
Occlusal force	20 (8–25)	13 (2–22)	*p* < 0.001 *^a^
/pa/ ODK repetitions/s	6 (5.6–6.6)	5.8 (5.0–6.4)	*p* < 0.001 *^a^
/ta/ ODK repetitions/s	6 (5.4–6.6)	5.8 (5.0–6.4)	0.031 *^a^
/ka/ ODK repetitions/s	5.6 (5.0–6.2)	5.2 (4.4–6)	*p* < 0.001 *^a^
Tongue pressure, kPa	33.3 (28.2–38.8)	28.9 (22.9–38.2)	0.007 *^a^
Masticatory function	123 (20.2)	12 (17.1)	0.534 ^b^
Swallowing function	0 (0–2)	0 (0–0.3)	0.030 *^a^

^a^ Mann–Whitney U-test, ^b^ Chi-square test, * *p* < 0.05. IQR: interquartile range; and ODK: oral diadochokinesis.

**Table 3:** Binomial logistic regression analysis: risk factors of oral function for cognitive impairment.

Variable		Univariate		Multivariate	
		OR (95%CI)	*p*-Value	OR (95%CI)	*p*-Value
Age, yrs		1.10 (1.06–1.14)	*p* < 0.001 *	1.11 (1.06–1.15)	*p* < 0.001 *
Sex		2.06 (1.26–3.40)	0.004 *	2.43 (1.42–4.16)	*p* < 0.001 *
BMI, kg/m^2^		1.03 (0.96–1.11)	0.413		
Education, yrs		1.08 (0.90–1.30)	0.425		
Smoking history, yrs		1.02 (1.006–1.032)	0.004 *		
Prescription drugs, n		1.09 (1.02–1.17)	0.014 *		
Solitary life		1.37 (0.81–2.33)	0.245		
Systemic disease	Stroke	1.94 (0.78–4.87)	0.157		
	Depression	3.36 (0.87–12.96)	0.079	4.60 (1.10–19.2)	0.036 *
SBP, mmHg		1.00 (0.98–1.01)	0.573		
DBP, mmHg		1.00 (0.97–1.02)	0.779		
HbA1c, %		1.22 (0.79–1.87)	0.373		
Oral hygiene		1.01 (1.00–1.01)	0.154		
Oral dryness		1.00 (0.91–1.12)	0.942		
Occlusal force		0.96 (0.94–0.98)	*p* < 0.001 *		
/pa/ ODK repetitions/s		0.62 (0.49–0.78)	*p* < 0.001 *	0.76 (0.59–0.96)	0.024 *
/ta/ ODK repetitions/s		0.68 (0.54–0.87)	0.002 *		
/ka/ ODK repetitions/s		0.63 (0.51–0.79)	*p* < 0.001 *		
Tongue pressure		0.96 (0.94–0.99)	0.005 *		
Masticatory function		0.82 (0.43–1.57)	0.541		
Swallowing function		0.95 (0.85–1.07)	0.384		

* *p* < 0.05. BMI: body mass index; CI: confidence interval; DBP: diastolic blood pressure; ODK: oral diadochokinesis; OR: odds ratio; and SBP: systolic blood pressure.

## Data Availability

The data presented herein are available upon request from the corresponding author.

## References

[1] Nagashima F., Furuse J. (2022). Treatments for elderly cancer patients and reforms to social security systems in Japan. Int. J. Clin. Oncol..

[2] Hosokawa R., Ojima T., Myojin T., Kondo K., Kondo N. (2023). Geriatric symptoms associated with healthy life expectancy in older people in Japan. Environ. Health Prev. Med..

[3] Tsuji I. (2020). Epidemiologic research on healthy life expectancy and proposal for its extension: A revised English version of Japanese in the Journal of the Japan Association 2019;148 (9):1781-4. JMA J..

[4] Takahashi T., Hatta K., Ikebe K. (2023). Risk factors of cognitive impairment: Impact of decline in oral function. Jpn. Dent. Sci. Rev..

[5] Nakahori N., Sekine M., Yamada M., Tatsuse T., Kido H., Suzuki M. (2021). Future projections of the prevalence of dementia in Japan: Results from the Toyama Dementia Survey. BMC Geriatr..

[6] Arvanitakis Z., Shah R.C., Bennett D.A. (2019). Diagnosis and management of dementia: Review. JAMA.

[7] Gauthier S., Reisberg B., Zaudig M., Petersen R.C., Ritchie K., Broich K., Belleville S., Brodaty H., Bennett D., Chertkow H. (2006). Mild cognitive impairment. Lancet.

[8] Anderson N.D. (2019). State of the science on mild cognitive impairment (MCI). CNS Spectr..

[9] Livingston G., Sommerlad A., Orgeta V., Costafreda S.G., Huntley J., Ames D., Ballard C., Banerjee S., Burns A., Cohen-Mansfield J. (2017). Dementia prevention, intervention, and care. Lancet.

[10] Livingston G., Huntley J., Sommerlad A., Ames D., Ballard C., Banerjee S., Brayne C., Burns A., Cohen-Mansfield J., Cooper C. (2020). Dementia prevention, intervention, and care: 2020 report of the Lancet Commission. Lancet.

[11] Nagatani M., Tanaka T., Son B.K., Kawamura J., Tagomori J., Hirano H., Shirobe M., Iijima K. (2023). Oral frailty as a risk factor for mild cognitive impairment in community-dwelling older adults: Kashiwa study. Exp. Gerontol..

[12] Tanaka K., Utsunomiya H., Kato H., Ogawa S., Suzuki H., Fujiwara Y., Nobuhara T., Senba H., Kimura E., Matsuura B. (2024). Association between tongue pressure and prevalence of mild cognitive impairment in Japan. Int. J. Geriatr. Psychiatry.

[13] Nakamura M., Hamada T., Tanaka A., Nishi K., Kume K., Goto Y., Beppu M., Hijioka H., Higashi Y., Tabata H. (2021). Association of oral hypofunction with frailty, sarcopenia, and mild cognitive impairment: A cross-sectional study of community-dwelling Japanese older adults. J. Clin. Med..

[14] Mitchell A.J., Shukla D., Ajumal H.A., Stubbs B., Tahir T.A. (2014). The Mini-Mental State Examination as a diagnostic and screening test for delirium: Systematic review and meta-analysis. Gen. Hosp. Psychiatry.

[15] Borson S., Scanlan J., Brush M., Vitaliano P., Dokmak A. (2000). The mini-cog: A cognitive ‘vital signs’ measure for dementia screening in multi-lingual elderly. Int. J. Geriatr. Psychiatry.

[16] Borson S., Scanlan J.M., Watanabe J., Tu S.-P., Lessig M. (2006). Improving identification of cognitive impairment in primary care. Int. J. Geriatr. Psychiatry.

[17] Tsoi K.K., Chan J.Y., Hirai H.W., Wong S.Y., Kwok T.C. (2015). Cognitive tests to detect dementia: A systematic review and meta-analysis. JAMA Intern Med..

[18] Borson S., Scanlan J.M., Chen P., Ganguli M. (2003). The Mini-Cog as a screen for dementia: Validation in a population-based sample. J. Am. Geriatr. Soc..

[19] Doerflinger D.M.C. (2007). How to try this: The mini-cog. Am. J. Nurs..

[20] Minakuchi S., Tsuga K., Ikebe K., Ueda T., Tamura F., Nagao K., Furuya J., Matsuo K., Yamamoto K., Kanazawa M. (2018). Oral hypofunction in the older population: Position paper of the Japanese Society of Gerodontology in 2016. Gerodontology.

[21] Shimizu T., Ueda T., Sakurai K. (2007). New method for evaluation of tongue-coating status. J. Oral Rehabil..

[22] Yamada H., Nakagawa Y., Nomura Y., Yamamoto K., Suzuki M., Watanabe N., Saito I., Seto K. (2005). Preliminary results of moisture checker for Mucus in diagnosing dry mouth. Oral Dis..

[23] Fukushima Y., Yoda T., Kokabu S., Araki R., Murata T., Kitagawa Y., Omura K., Toya S., Ito K., Funayama S. (2013). Evaluation of an oral moisture-checking device for screening dry mouth. Open J. Stomatol..

[24] Gotfredsen K., Walls A.W. (2007). What dentition assures oral function?. Clin. Oral Implant. Res..

[25] Yamada A., Kanazawa M., Komagamine Y., Minakuchi S. (2015). Association between tongue and lip functions and masticatory performance in young dentate adults. J. Oral Rehabil..

[26] Watanabe Y., Hirano H., Arai H., Morishita S., Ohara Y., Edahiro A., Murakami M., Shimada H., Kikutani T., Suzuki T. (2017). Relationship between frailty and oral function in community-dwelling elderly adults. J. Am. Geriatr. Soc..

[27] Tanaka Y. (2015). Examination about the relation of meal form, tongue pressure, grip and walking state in inpatient and elderly residents. Jpn. J. Dysphagia Rehabil..

[28] Tanaka T., Takahashi K., Hirano H., Kikutani T., Watanabe Y., Ohara Y., Furuya H., Tetsuo T., Akishita M., Iijima K. (2018). Oral frailty as a risk factor for physical frailty and mortality in community-dwelling elderly. J. Gerontol. B.

[29] Belafsky P.C., Mouadeb D.A., Rees C.J., Pryor J.C., Postma G.N., Allen J., Leonard R.J. (2008). Validity and reliability of the Eating Assessment Tool (EAT-10). Ann. Otol. Rhinol. Laryngol..

[30] Schindler A., de Fátima Lago Alvite M., Robles-Rodriguez W.G., Barcons N., Clavé P. (2023). History and science behind the Eating Assessment Tool-10 (Eat-10): Lessons Learned. J. Nutr. Health Aging.

[31] Vatcheva K.P., Lee M., McCormick J.B., Rahbar M.H. (2016). Multicollinearity in regression analyses conducted in epidemiologic studies. Epidemiology.

[32] Fiorillo L. (2019). Oral health: The first step to well-being. Medicina.

[33] Scannapieco F.A. (2023). Poor oral health in the etiology and prevention of aspiration pneumonia. Clin. Geriatr. Med..

[34] Nativ-Zeltzer N., Nachalon Y., Kaufman M.W., Seeni I.C., Bastea S., Aulakh S.S., Makkiyah S., Wilson M.D., Evangelista L., Kuhn M.A. (2022). Predictors of aspiration pneumonia and mortality in patients with dysphagia. Laryngoscope.

[35] Manabe T., Fujikura Y., Mizukami K., Akatsu H., Kudo K. (2019). Pneumonia-associated death in patients with dementia: A systematic review and meta-analysis. PLoS ONE.

[36] Sakakibara R., Iimura A., Ogata T., Terayama K., Katsuragawa S., Nagao T., Suzuki K., Izawa K., Nakajima K., Haruki S. (2022). Brain diseases and aspiration pneumonia in older person. Neurol. Clin. Neurosci..

[37] Funayama M., Koreki A., Takata T., Hisamatsu T., Mizushima J., Ogino S., Kurose S., Oi H., Mimura Y., Shimizu Y. (2023). Pneumonia risk increased by dementia-related daily living difficulties: Poor oral hygiene and dysphagia as contributing factors. Am. J. Geriatr. Psychiatry.

[38] Pandya S.Y., Lacritz L.H., Weiner M.F., Deschner M., Woon F.L. (2017). Predictors of reversion from mild cognitive impairment to normal cognition. Dement. Geriatr. Cogn. Disord..

[39] Ellendt S., Voβ B., Kohn N., Wagels L., Goerlich K.S., Drexler E., Schneider F., Habel U. (2017). Predicting stability of mild cognitive impairment (MCI): Findings of a community based sample. Curr. Alzheimer Res..

[40] Koepsell T.D., Monsell S.E. (2012). Reversion from mild cognitive impairment to normal or near-normal cognition: Risk factors and prognosis. Neurology.

[41] Padmanabhan H., Vijayakumar S.S., Kumar V.A. (2022). Comparison of the effect of conventional and implant-retained overdentures on brain activity and cognition in a geriatric population-A functional MRI study. J. Prosthodont. Res..

[42] FFujiwara Y., Suzuki H., Yasunaga M., Sugiyama M., Ijuin M., Sakuma N., Inagaki H., Iwasa H., Ura C., Yatomi N. (2010). Brief screening tool for mild cognitive impairment in older Japanese: Validation of the Japanese version of the Montreal Cognitive Assessment. Geriatr. Gerontol. Int..

[43] Makizako H., Shimada H., Park H., Doi T., Yoshida D., Uemura K., Tsutsumimoto K., Suzuki T. (2013). Evaluation of multidimensional neurocognitive function using a tablet personal computer: Test-retest reliability and validity in community-dwelling older adults. Geriatr. Gerontol. Int..

[44] Li X., Dai J., Zhao S., Liu W., Li H. (2018). Comparison of the value of Mini-Cog and MMSE screening in the rapid identification of Chinese outpatients with mild cognitive impairment. Medicine.

[45] Folstein M.F., Folstein S.E., McHugh P.R. (1975). “Mini-mental state”. A practical method for grading the cognitive state of patients for the clinician. J. Psychiatr. Res..

[46] Anthony J.C., LeResche L., Niaz U., von Korff M.R., Folstein M.F. (1982). Limits of the ‘Mini-Mental State’ as a screening test for dementia and delirium among hospital patients. Psychol. Med..

[47] Palsetia D., Rao G.P., Tiwari S.C., Lodha P., De Sousa A. (2018). The clock drawing test versus mini-mental status examination as a screening tool for dementia: A clinical comparison. Indian J. Psychol. Med..

[48] Yajima S., Nakanishi Y., Matsumoto S., Ookubo N., Tanabe K., Kataoka M., Masuda H. (2022). The Mini-Cog: A simple screening tool for cognitive impairment useful in predicting the risk of delirium after major urological cancer surgery. Geriatr. Gerontol. Int..

[49] Tiwary N., Treggiari M.M., Yanez N.D., Kirsch J.R., Tekkali P., Taylor C.C., Schenning K.J. (2021). Agreement between the Mini-Cog in the preoperative clinic and on the day of surgery and association with postanesthesia care unit delirium: A cohort study of cognitive screening in older adults. Anesth. Analg..

[50] Saito H., Yamashita M., Endo Y., Mizukami A., Yoshioka K., Hashimoto T., Koseki S., Shimode Y., Kitai T., Maekawa E. (2020). Cognitive impairment measured by Mini-Cog provides additive prognostic information in elderly patients with heart failure. J. Cardiol..

[51] Abayomi S.N., Sritharan P., Yan E., Saripella A., Alhamdah Y., Englesakis M., Tartaglia M.C., He D., Chung F. (2024). The diagnostic accuracy of the Mini-Cog screening tool for the detection of cognitive impairment-A systematic review and meta-analysis. PLoS ONE.

[52] Lorentz W.J., Scanlan J.M., Borson S. (2002). Brief screening tests for dementia. Can. J. Psychiatry.

[53] Langa K.M., Levine D.A. (2014). The diagnosis and management of mild cognitive impairment: A clinical review. JAMA.

[54] Watanabe Y., Arai H., Hirano H., Morishita S., Ohara Y., Edahiro A., Murakami M., Shimada H., Kikutani T., Suzuki T. (2018). Oral function as an indexing parameter for mild cognitive impairment in older adults. Geriatr. Gerontol. Int..

[55] Ostrand R., Gunstad J. (2021). Using automatic assessment of speech production to predict current and future cognitive function in older adults. J. Geriatr. Psychiatry Neurol..

[56] Savarimuthu A., Ponniah R.J. (2023). A Slip between the brain and the lip: Working memory and cognitive-communication disorders. J. Psycholinguist Res..

[57] Sakurai R., Fujiwara Y., Ishihara M., Higuchi T., Uchida H., Imanaka K. (2013). Age-related self-overestimation of step-over ability in healthy older adults and its relationship to fall risk. BMC Geriatr..

[58] Fu Y., Luo X., Zeng Q., Li K., Zhang T., Li Z., Xu X., Hong L., Chen Y., Zhang M. (2021). Effects of anosognosia on static and dynamic amplitudes of low-frequency fluctuation in mild cognitive impairment. Front. Aging Neurosci..

